# An Emerging Conundrum in Nervous System Infections: Balancing Virus Offense and Host Defense

**DOI:** 10.3390/v15102071

**Published:** 2023-10-10

**Authors:** Lynn Enquist

**Affiliations:** Department of Molecular Biology, Princeton University, Princeton, NJ 08540, USA; lenquist@princeton.edu

Most viral infections begin in the peripheral organs and, as a result, engage the circulatory (blood and lymph) system, releasing more virus particles and other products of the infection (both host and virus gene products) into the circulation. Occasionally, some viral infections spread directly to the peripheral nervous system (PNS). Peripheral lesions and inflammation are common symptoms of viral infections. Some viruses can infect many cell types, while others infect only one subset. Both phenotypes reflect the presence or absence of virus-specific receptors. It is well known that viral infections vary in their capacities to establish productive infections in their hosts. This phenomenon reflects the accessibility to susceptible cells, the extent of host defenses, and the action of viral gene products that modulate, bypass, or otherwise mediate the escape from host defenses. It is a truism that all viral genomes express a variety of gene products that modulate host defenses so that a productive infection can be established. These host defensive actions vary across individuals and tissues due in part to age, immune capacity, and general health. Both host genetics and their epigenetic state also play central roles in the efficiency of viral infections and the establishment of infections in a host population.

Progress in understanding how viral offense and host defense engage each other has been significant. However, most of the progress has been at the cellular level, and not at the organ or organ system levels. A conundrum concerning peripheral infections, and the subsequent response of the nervous system, is becoming apparent: despite a barrage of peripheral organ infections over the lifetime of a host, the brain and spinal cord (which comprise the central nervous system; CNS) are rarely infected directly, but often respond indirectly. There are noteworthy exceptions. The rabies virus and its relatives usually spread from the periphery to the CNS with high efficiency. Some enteroviruses, such as poliovirus, usually stay in the periphery, but can also cause serious CNS disease in a minority of infected individuals. Recent work suggests that some common RNA virus infections (e.g., flaviviruses and coronaviruses) may have both acute CNS effects (encephalitis) and long-term consequences, such as neurodegeneration or peripheral neuropathies. These latter effects most likely represent indirect responses to peripheral infections, but the details are often lacking. For example, the penetrance of neurological symptoms is not 100%. How can a peripheral infection involviing many people cause neuropathogenic effects in only a few individuals?

We know that all peripheral infections have indirect effects on CNS function. Indeed, the common symptoms of most infections (fever, aches, fatigue, stomach problems) reflect a CNS response in one way or another. The common symptoms are usually caused by indirect effects from inflammatory cytokines released to the blood from the infected peripheral tissues. It is increasingly clear that overproduction or uncontrolled expression of these cytokines can have devastating effects, but the effects of low-level chronic inflammation after acute infections are not always obvious. The effects may not all be due to the hematogenous delivery of bioreactive molecules to the CNS, but may reflect a more global PNS response to the inflamed tissues.

The PNS can be directly infected after peripheral infection by some viruses. Most of the information about the PNS and viral infections centers around infections by alpha herpesviruses, which always spread from peripheral organ infection to the PNS that innervates that tissue. A quiescent infection is often established in these PNS neurons. In fact, most of the world’s human population carries an alpha herpesvirus genome in their PNS. Remarkably, these infected PNS neurons still function reasonably well. After a global stress response, a quiescent PNS infection can reactivate in some (but not all) neurons, producing virus particles that spread back to the original site of infection. The threshold for this reactivation is high. In this way, the alpha herpesviruses promote transmission to other hosts, and also maintain the function of the nervous system. Even in these reactivating PNS infections, their spreading to the CNS is rare in healthy individuals. An understudied issue is how the PNS retains function despite being infected; another is why some people suffer CNS infections while most do not.

The response of the PNS to peripheral infections is poorly understood. Each peripheral PNS ganglia, while an independent signaling unit, is likely to inform other ganglia of local insults, in order to maintain the homeostasis of the system. In addition, the enteric nervous system, a quasi-autonomous part of the nervous system, receives input from the gastrointestinal tract and external input from the CNS. Its function is also modulated by the PNS, including the parasympathetic vagus nerve, which innervates most of the viscera. Little is known about how the enteric nervous system is affected by viral infections.

The beta and gamma herpesviruses may provide more insights into indirect effects of viral infections on the nervous system. Like the alpha herpesviruses, both beta and gamma herpesviruses establish lifelong quiescent infections in most people. These viruses rarely infect the PNS or CNS directly, but rather infect the cells of the hematopoietic system (beta herpesviruses) or the B and T cells of the immune system (gamma herpesviruses). Nevertheless, beta herpesvirus infections of pregnant women can cause frequent fetal hearing loss and aberrant fetal brain development. By contrast, immune system defects promoted by gamma herpesvirus infections can cause rare autoimmune neurodegenerative diseases.

The CNS is one of the most protected tissues in the host, while the PNS is more exposed. There is a significant barrier to the spread of virus particles and many proteins to the CNS from the circulatory system: the so called blood–brain barrier. CNS infections that circumvent the blood–brain barrier often reflect a breakdown in host defense, either due to powerful viral offensive gene products, to reduced host defenses, or both. By contrast, PNS ganglia are surveilled regularly by T and B cells in the periphery, and are routinely exposed to inflammatory cytokines produced by infected and innervated tissues. Perhaps surprisingly, the PNS can withstand the direct and indirect effects of many acute infections without losing functionality. Support cells, such as glia and other satellite cells, certainly provide powerful protection. The effect of long-term exposure of the PNS to peripheral inflammation on the host is not well known.

Clearly, terminally differentiated neurons in general, unlike most non-neuronal cells, do not engage cell death or apoptotic pathways when exposed to virus infection. This phenotype may reflect fact that most neurons are irreplaceable, and essential neuronal circuitry cannot be interrupted. As a result, new modes of nervous system cellular defenses have evolved that are only now being appreciated. In general, despite the fact they are intimately engaged with peripheral organs, both the brain and spinal cord are protected from severe peripheral insults.

Indeed, there is much we do not know. The challenge is to understand the direct and indirect mechanisms of action between gene products of viral offenses and host defenses. The emerging conundrum of balancing virus offense with nervous system defense after acute and persistent infection will be resolved when we understand how the CNS and PNS command and control systems respond to infection while remaining protected. At a minimum, we must understand the effects of chronic (persistent) infections, long term (fluctuating level) exposure of the PNS and CNS to inflammatory cytokines, and variable host defenses in the host population.

